# Occurrence of methicillin-resistant staphylococci in the pig-production chain in Ibadan, Nigeria

**DOI:** 10.4102/ojvr.v88i1.1959

**Published:** 2021-11-30

**Authors:** Opeyemi U. Lawal, Abimbola O. Adekanmbi, Olawale O. Adelowo

**Affiliations:** 1 Department of Microbiology, Faculty of Science, University of Ibadan, Ibadan, Nigeria; 2 Canadian Research Institute for Food Safety (CRIFS), Department of Food Science, Ontario Agricultural College, University of Guelph, Guelph, Ontario, Canada

**Keywords:** methicillin-resistant staphylococci, pigs, pork samples, colonisation, food contamination, food chain

## Abstract

*Staphylococcus* species colonises humans and animals and is a major food contaminant with public health significance. Here, we assessed the occurrence of methicillin-resistant staphylococci (MRS) in the pig-production chain in Ibadan, Nigeria. Nares of 120 pigs and 10 farmers were sampled with sterile swabs whilst 54 pork samples were collected from a retail slaughterhouse. *Staphylococcus* species were isolated using enrichment, cefoxitin–aztreonam selective broth and Mannitol salt agar. Isolates were tested for susceptibility to cefoxitin (30 μg), oxacillin (1 μg) and vancomycin (30 μg). Methicillin-resistant staphylococci isolates were characterised using conventional biochemical tests. From 184 samples, 364 staphylococcal isolates were obtained. Amongst the 54 pork samples, 44.0% were contaminated with *Staphylococcus* species. Overall, 9 (2.5%) MRS were obtained and presumptively identified as *Staphylococcus xylosus* (*n* = 3), *Staphylococcus sciuri* (*n* = 3), *Staphylococcus warneri* (*n* = 2) and *Staphylococcus cohnii* (*n* = 1). There was no relationship between the prevalence of MRS between pigs and pig handlers in the farms, but Farm 2 had the highest frequency of 66.7% (*p* < 0.05). Piglets had the highest prevalence of 66.7% (*p* < 0.05) whilst MRS was absent in workers and pork samples. This study raises concerns about the cross-contamination of staphylococci in the food chain. Constant surveillance is imperative to ensure food safety.

## Background

*Staphylococcus* species colonises humans and animals and is one of the major food contaminants of public health importance (Becker, Heilmann & Peters 2014). One of the challenges for controlling these bacteria is their development of resistance to antimicrobials including methicillin. Methicillin-resistant staphylococci (MRS) emerged in a variety of livestock animals, apparently through different mechanisms (De Boer et al. 2009). The close proximity amongst humans, livestock and antimicrobial use in animals presumably facilitates the emergence and spread of MRS (Bouchami et al. 2020). Methicillin-resistant staphylococci have been recovered from various retail products (Bouchami et al. 2020; De Boer et al. 2009) leading to an interest in MRS colonisation and infection in animals. Studies have shown that MRS could be transmitted between pigs (Verhegghe et al. 2013) and from pigs to their handlers (Bouchami et al. 2020). Actually, piglets are reported to be more vulnerable to MRS colonisation and infection because of the fragility of their immune system (Verhegghe et al. 2013).

Pork is one of the most consumed food commodities globally. Contamination of pork by staphylococci or MRS could result from cross-contamination from colonised pigs before slaughter or within the slaughterhouse by handlers that could end up in the food chain. However, handling contaminated meat could increase the risk of colonisation and infection in immunocompromised individuals (Bouchami et al. 2020). This study aimed to assess the contamination rate of retail pork samples and the occurrence of MRS along the pig-production chain in Ibadan, southwestern Nigeria.

## Methods

### Description of the study site

The study areas included five farms selected based on their proximity to densely populated communities located within the city of Ibadan, Oyo state, Nigeria. We employed a convenience sampling strategy wherein at least 30% of all categories of pigs and pig farmers resident in each farm at a time were sampled. Moreover, meats from an open-air slaughterhouse at Bodija market, Ibadan, where an average of 20 pigs per day are slaughtered were sampled (Table 1).

**TABLE 1:** Summary of the sampling, antimicrobial use, *Staphylococcus species,* and methicillin-resistant isolates recovered in this study.

Sample sites	Location	Antimicrobial use	Herd size	No of sample	No positive samples	No of staphylococcal isolates	No of MRS carrier	*Presumptive* MRS species
Farm 1	7° 23’ 11” N,	No record	Pigs: 42	Pigs: 15	16	36	2	*Staphylococcus xylosus* (*n* = 2)
	3° 50’ 1”E		Workers: 2	Workers: 1				
Farm 2	7° 27’ 18”N,	Immunosol, levamisol, nivamectin, envit, oxytetracycline, pens trep, tylophan, oxime, pemacom	Pigs > 200	Pigs: 60	67	154	6	*Staphylococcus sciuri* (*n* = 3),
	3° 53’ 46” E		Workers: > 10	Workers: 7				*Staphylococcus warneri* (*n* = 2)
								*Staphylococcus cohnii* (*n* = 1)
Farm 3	7° 38’ 36” N,	Oxime LA, B complex Iron injection and Ivomec	Pigs: 15	Pigs: 15	15	30	None	None
	4° 43’ 13” E		Workers 4	Workers 0				
Farm 4	7° 23’ 19” N,	No record	Pigs: 40	Pigs: 15	15	30	None	None
	3° 50’ 7” E		Workers: 3	Workers 0				
Farm 5	7° 22’ 25.05” N,	Oxime LA and Ivomec	Pigs: 42	Pigs: 15	17	42	1	*Staphylococcus xylosus*
	3° 50’ 48.63” E		Workers: 4	Workers: 2				
Pork samples	7° 26’ 15”N,	NA	20 pigs/day	54	24	72	None	None
	3° 55’ 24” E							

**Total**	**NA**	**NA**	**NA**	**184**	**154**	**364**	**9**	**NA**

NA, not applicable; MRS, methicillin-resistant staphylococci.

### Sample collection

Nares of 120 pigs and 10 pig handlers were sampled using a sterile swab. Pork samples were collected once every fortnight from 10 pigs for five weeks. A total of 140 nasal swabs and 54 pork samples were collected. Samples were transported on ice to the laboratory for analysis (Table 1).

### Bacterial isolation and characterisation

Isolation was performed as described previously (Vestergaard et al. 2012) using a three-step procedure. Briefly, swab sticks and 25 g of meat sample were inoculated in 225 mL peptone water (Oxoid) supplemented with 10 mL 6.5% NaCl and incubated at 37 °C for 18–24 h. A 1 mL overnight culture was transferred unto tryptone soya broth (TSB, Oxoid) supplemented with 4 mg/L cefoxitin and 75 mg/L aztreonam and incubated at 37 °C for 18–24 h. A total of 10 μL overnight TSB culture was streaked on mannitol salt agar (Oxoid) and incubated at 37 °C for 24–48 h. The distinct, creamy to yellow colonies showing halo zones, which represent presumptive mannitol fermenting staphylococcal isolates, were subjected to preliminary biochemical tests, namely *Gram* staining, catalase, oxidase, DNase, blood haemolysis, urease production, l-arginine, gelatin hydrolysis, sugar fermentation and tube coagulase tests, as well as osmotolerance assays.

### Antibiotics susceptibility testing

This was determined by the disk diffusion method according to Clinical and Laboratory Standards Institute (CLSI) guideline (CLSI 2015). Briefly, three colonies from an overnight culture were suspended in 5 mL of sterile normal saline. Optical density was adjusted to 0.5 McFarland standard, and sterile swab sticks were used to evenly spread the inocula over the entire surface of Mueller-Hilton Agar (Oxoid) plates supplemented with 4% sodium chloride. Antibiotic disks, namely novobiocin (5 μg), cefoxitin (30 μg), oxacillin (1 μg), and vancomycin (30 μg), were placed on the Mueller-Hilton agar plate and incubated at 37 °C for 24 h. The diameter of the zone of growth inhibition around each disc was measured, and the results were interpreted using the CLSI guideline (CLSI 2015).

### Statistical analysis

Descriptive analyses were initially performed. Microsoft Excel Spreadsheet was used for data processing. Frequency of MRS amongst groups was compared using one-way analysis of variance (ANOVA) with Bonferroni post-test using GraphPad InStat Version 3.00 for Windows 95. Association was deemed significant at *p* < 0.05.

### Ethical considerations

Ethical approval was obtained from the University of Ibadan review board (reference: UI/EC/13/0332).

## Results

### Study farms and antimicrobial usage

The five farms selected for this study were involved in pig production but lack accurate history of the type and quantity of antimicrobials used on the farms. Although the farm workers admitted to a regular use of antimicrobial as a feed additive and as prophylaxis, only three farms provided the necessary information to confirm this. Farm 2 uses immunosol (an immunomodulator), nivamectin (an antiparasitic) and oxytetracycline (an antibiotic), whereas Farms 3 and 5 use oxime LA, ivomec (antiparasitic) and B complex iron injection (Table 1).

### High contamination rate of the pig production chain by *Staphylococcus* species

Using three-step method, all the samples recovered from pigs and pig handlers were positive for mannitol-positive *Staphylococcus* species. This is not surprising because the sampled site is the ecological niche for some of the staphylococcal species. However, 44% (*n* = 24/54) of the pork samples were contaminated with mannitol-positive *Staphylococcus* species. Overall, 364 staphylococcal isolates were recovered from 184 samples (Table 1).

### Low occurrence of methicillin resistance amongst staphylococcal isolates recovered in pig-production chain

**FIGURE 1:**
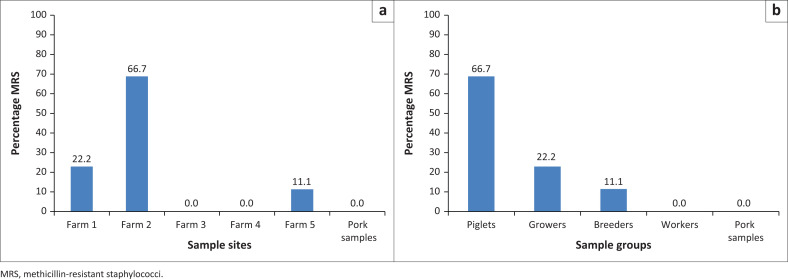
Occurrence of methicillin-resistant staphylococci (MRS) along the pig-production chain in Ibadan. (a) Distribution of methicillin-resistant staphylococci in different sample sites. (b) Distribution of methicillin-resistant staphylococci in different sample groups.

In spite of using cefoxitin (4 mg/L) to select for MRS, the antibiotics susceptibility testing yielded a low prevalence of MRS, as only 9 isolates (2.5%, 9/364) were methicillin resistant (Table 1). These isolates were obtained from nine different pigs from three farms suggesting an overall MRS pig colonisation rate of 7.5% (*n* = 9/120; Table 2). Methicillin-resistant staphylococci were not recovered from workers or pork samples, and none of the isolates was found to be resistant to vancomycin. MRS were presumptively identified using biochemical tests as four coagulase-negative staphylococcal species, namely *Staphylococcus sciuri* (*n* = 3), *Staphylococcus xylosus* (*n* = 3), *Staphylococcus warneri* (*n* = 2) and *Staphylococcus cohnii* (*n* = 1; Table 2). It is noteworthy that the great majority of the MRS were recovered from Farm 2 (*n* = 6, 66.7%, *p* < 0.05) and from piglets (*n* = 6, 66.7%, *p* < 0.05). No MRS was obtained from Farms 3 and 4 and from pork samples (Figure 1; Table 1).

**TABLE 2:** Distribution of methicillin-resistant staphylococci among the sample groups.

Sample groups	No of samples	No of staphylococcal isolates	No of MRS carrier	*Presumptive* MRS species
Piglets	50	95	6	*Staphylococcus cohnii* (*n* = 1), *Staphylococcus xylosus* (*n* = 1),
				*Staphylococcus sciuri* (*n* = 2), *Staphylococcus warneri* (*n* = 2)
Growers	50	97	2	*Staphylococcus sciuri, Staphylococcus xylosus*
Breeders	20	40	1	*Staphylococcus xylosus*
Workers	10	60	None	None
Pork samples	54	72	None	None

MRS, methicillin-resistant staphylococci.

## Discussion

Microbial contamination along the food chain poses a great threat to food safety and public health. In this study, we assessed the contamination of pork samples in a community market in Ibadan, southwest Nigeria, and the occurrence of MRS along the pig-production chain. Only nine samples were positive for MRS, all of which were from pigs, suggesting an overall MRS pig colonisation rate of 7.5%, a value that is comparable to previous studies (Peng et al. 2019). Contrarily, a study (Verstappen et al. 2017) reported a higher prevalence of MRS (20%) in livestock pigs. The great majority of the MRS (*n* = 6/9) were from piglets, an observation that reiterates piglets to have the propensity to harbour antibiotic-resistant bacteria because of their immune system fragility (Verhegghe et al. 2013). Alternatively, the sow could be the source of these MRS pre-weaning period or possibly may be acquired from the environment.

Surprisingly, the antimicrobial used in the farms studied were mostly similar and amongst those recommended for use in animal husbandry (Doyle, Hartmann & Wong 2011). Whilst, the use of oxytetracycline in animal husbandry is reported to drive increased antimicrobial resistance, particularly MRS (Doyle et al. 2011), we could not establish this link in the study.

The overall frequency of MRS was low, as only 2.5% of the 364 mannitol-fermenting *Staphylococcus* species obtained were MRS. All the MRS isolates obtained were coagulase negative whilst none was found to be coagulase positive. This could be as a result of competition for site colonisation amongst staphylococci found in such environment (Tulinski et al. 2012). Amongst the nine MRS recovered, the most common species were *S. sciuri* (*n* = 3) *and S. xylosus* (*n* = 3), whilst others were *S. warneri* (*n* = 2) and *S. cohnii* (*n* = 1). A similar study conducted in pig farms also recovered methicillin-resistant strains of these species in addition to *Staphylococcus lentus* (Rattanamuang, Butr-Indr & Anukool 2013). *Staphylococcus sciuri* colonises the skin and nares of humans and animals (Shittu et al. 2006) and is associated with human infections including urinary tract infections (UTI), endocarditis, peritonitis and wound infections (Couto et al. 2000). It could also be implicated in fatal exudative epidermitis in piglets (Chen et al. 2007). *Staphylococcus warneri* colonises the skin of both animals and humans and occasionally causes septicaemia, endocarditis, osteomyelitis and other types of infections occurring in immunocompromised individual or patients with invasive treatment procedure. In humans, *S. xylosus* could be implicated in UTI and more rarely, in endocarditis, pyelonephritis or pneumonia (Becker et al. 2014). *Staphylococcus cohnii* is an opportunistic pathogen found colonising human skin in only small numbers. The colonisation of *S. cohnii* in hospital environments has been previously reported, with almost all isolates identified as methicillin resistant (Heilmann, Ziebuhr & Becker 2019).

Despite the absence of MRS in the retail pork samples investigated, the high contamination of these products by mannitol-positive staphylococci (44%) could be of public health concern. This rate is comparable to those reported previously (Bouchami et al. 2020). Understandably, most staphylococci are part of human normal flora (Becker et al. 2014), but their presence in retail meat products suggests a cross-contamination phenomenon, most likely from human or the environment. Hence, a stricter hygienic programme would be appropriate to enforce and promote food safety practices.

## Conclusion

This report assessed the antimicrobial use, occurrence of MRS in pig farms and explored the extent of contamination of retail pork samples. Although this study showed a low occurrence of MRS in the samples studied, the high contamination of pork samples by *Staphylococcus* species is of great concern. Proper monitoring and surveillance programmes are recommended for the early detection of staphylococcal foodborne outbreaks.
